# Anti-Inflammatory and Immunomodulatory Properties of a Crude Polysaccharide Derived from Green Seaweed *Halimeda tuna*: Computational and Experimental Evidences

**DOI:** 10.3390/md22020085

**Published:** 2024-02-11

**Authors:** Marwa Kraiem, Sonia Ben Hamouda, Malek Eleroui, Marwa Ajala, Amal Feki, Amel Dghim, Zakaria Boujhoud, Marwa Bouhamed, Riadh Badraoui, Jean Marc Pujo, Khadija Essafi-Benkhadir, Hatem Kallel, Ibtissem Ben Amara

**Affiliations:** 1 Laboratory of Medicinal and Environment Chemistry, Higher Institute of Biotechnology, University of Sfax, PB 261, Sfax 3000, Tunisia; marwakrayem92@gmail.com (M.K.); aroui.malek@gmail.com (M.E.); ajalamarwa1996@gmail.com (M.A.); amal.feki05@gmail.com (A.F.); dsirine51@gmail.com (A.D.); 2 Laboratory of Molecular Epidemiology and Experimental Pathology–LR16IPT04, Pasteur Institute of Tunis, University of Tunis El Manar, Tunis 1002, Tunisia; soniabenhamoudaa@gmail.com (S.B.H.); khadija.essafi@pasteur.utm.tn (K.E.-B.); 3 Laboratory of Health Sciences and Technologies, Higher Institute of Health Sciences of Settat, Hassan First University of Settat, Settat 26000, Morocco; boujhoud.zak@gmail.com; 4 Laboratory of Anatomopathology, CHU Habib Bourguiba, University of Sfax, Sfax 3029, Tunisia; marwa4272@gmail.com; 5 Department of General Biology, University of Ha’il, Ha’il 81451, Saudi Arabia; riadh.badraoui@fmt.utm.tn; 6 Section of Histology–Cytology, Medicine Faculty of Tunis, University of Tunis El Manar, La Rabta 1007, Tunisia; 7 Emergency Department, Cayenne General Hospital, Cayenne 97300, French Guiana; tamac1966@gmail.com; 8 Intensive Care Unit, Cayenne General Hospital, Cayenne 97300, French Guiana; kallelhat@yahoo.fr; 9 Tropical Biome and Immunopathology CNRS UMR-9017, Inserm U 1019, University of Guiana, Cayenne 97300, French Guiana

**Keywords:** crude polysaccharide, PSHT, anti-oxidant, anti-hemolytic, immunomodulatory, molecular interactions, RAW 264.7, anti-inflammatory activity, computational analysis

## Abstract

In this study, we investigated for the first time the anti-inflammatory and immunomodulatory properties of crude polysaccharide (PSHT) extracted from green marine algae *Halimeda tuna*. PSHT exhibited anti-oxidant activity in vitro through scavenging 1, 1-diphenyl-2-picryl hydroxyl free radical, reducing Fe^3+^/ferricyanide complex, and inhibiting nitric oxide. PSHT maintained the erythrocyte membrane integrity and prevented hemolysis. Our results also showed that PSHT exerted a significant anti-edematic effect in vivo by decreasing advanced oxidation protein products and malondialdehyde levels and increasing the superoxide dismutase and glutathione peroxidase activities in rat’s paw model and erythrocytes. Interestingly, PSHT increased the viability of murine RAW264.7 macrophages and exerted an anti-inflammatory effect on lipopolysaccharide-stimulated cells by decreasing pro-inflammatory molecule levels, including nitric oxide, granulocyte-macrophage colony-stimulating factor (GM-CSF) and tumor necrosis factor-alpha (TNF-α). Our findings indicate that PSHT could be used as a potential immunomodulatory, anti-inflammatory, anti-hemolytic, and anti-oxidant agent. These results could be explained by the computational findings showing that polysaccharide building blocks bound both cyclooxygenase-2 (COX-2) and TNF-α with acceptable affinities.

## 1. Introduction

Inflammation is a beneficial organism defense reaction against injurious agents [1,2]. However, the chronic and uncontrolled inflammatory response entail several disorders, including cancer, type 2 diabetes, rheumatoid arthritis, and cardiovascular diseases.

Macrophages, the central players in innate and adaptive immunity, play critical roles in the inflammatory response and tissue repair. They can enhance and/or reduce the host response by regulating the production of several effectors and mediators such as inflammatory cytokines, nitric oxide (NO), prostaglandins E (PGE), and reactive oxygen species (ROS) [3,4].

Several conventional drugs are used to control the inflammatory reaction [5]. However, their long-term use could be associated with gastrointestinal and cardiovascular side effects. Therefore, the search for alternative and nontoxic anti-inflammatory agents is of paramount importance.

Marine green algae are a source of natural bioactive compounds enriched with health-beneficial properties [6,7,8,9]. They contain a variety of secondary metabolites, such as polysaccharides, which are natural macromolecules with complex, critical, and multifaceted pharmacological activities, including anti-tumor, anti-oxidant, anti-inflammatory, and immunomodulatory properties [9,10,11]. In addition to their broad spectrum biological activities, they exhibit relatively low toxicity [12,13,14].

The evaluation of the viability of RAW 264.7 murine macrophages has been used as an indicator of macrophages activation and, subsequently, for the characterization of the immunomodulatory potential of new compounds with biomedical applications. Several studies highlighted the immunomodulatory functions of marine polysaccharides by inducing macrophage production of key immunomodulatory mediators such as NO, PGE, ROS, and macrophage colony-stimulating factor (M-CSF). A sulfate polysaccharide, called GLP-2, extracted from the red alga *Gracilaria lemaneiformis* [15], significantly improves the proliferation and pinocytic capacity of murine RAW264.7 macrophages.

The process of pinocytosis is essential for the cell well-being and provides a mechanism for the uptake and degradation of macromolecules. It contributes to the host’s immunocompetency in antigen uptake, internalization, and processing [15,16]. This enhancement is facilitated by the macrophage ability to stimulate the generation of ROS and NO. Qin et al. (2022) [17] reported that a sulfate polysaccharide DAP4 from brown alga *Durvillaea antarctica* exhibited excellent immunomodulatory activity by promoting spleen lymphocyte proliferation, increasing NO production, and improving macrophage phagocytic activity.

Modulating macrophage-mediated inflammatory response is pivotal for developing a therapeutic strategy for inflammatory diseases. Sulfated polysaccharides isolated from the green seaweed *Codium fragile* reduced the levels of PGE2 and NO in LPS-induced RAW 264.7 cells [11]. PGE2 is a significant cyclooxygenase (COX) product at inflammatory sites, where it contributes to local increase in blood flow, edema formation, and pain sensitization [18]. Thus, inhibiting these pro-inflammatory mediators would be an effective strategy for treating inflammatory diseases.

Furthermore, polysaccharides can prevent leukocyte migration to inflammation sites and modulate the production of numerous factors involved in the inflammatory process, including PGE2, NO, interleukin-1 beta (IL-1β), tumor necrosis factor-alpha (TNF-α), interleukin-6 (IL-6) cells, and interleukin-10 (IL-10). The biological function of carbohydrates identified in many marine algae against the inflammatory process is well defined [18,19].

*Halimeda* is a genus of warm, temperate-to-tropical calcareous macroalgae. Previous reports demonstrated various pharmacological properties, like antibacterial [20], anti-oxidant [21], antifungal [22], and hepatoprotective [23,24] effects, with specimens isolated from different oceanic regions. Despite its significance, few studies highlighted the bioactivities of *Halimeda tuna* marine algae extracts [25,26]. This alga remains unexploited, and there are still some challenges in this field. More importantly, nothing is known regarding its potential immunomodulatory and anti-inflammatory properties. Based on its potentially interesting biological activity, *Halimeda tuna* algae were tested for their biotechnological and pharmacological properties. Thus, the current research investigated whether polysaccharides extracted from *Halimeda tuna* could exhibit in vitro and in vivo anti-inflammatory properties and reduce hematological disorders and oxidative damage in both skin and erythrocytes. Computational modeling also studied the molecular interactions of the polysaccharide units with COX-2 and TNF-α, which are linked to the assessed activities.

## 2. Results and Discussion

### 2.1. Extraction Yield and Chemical Characterization

Polysaccharides are polar polymers that dissolve quickly in water due to their ability to replace water–water with water–solute interactions. Based on *H. tuna* dry weight, the extraction of PSHT yielded 15.9% ± 0.5 (*w*/*w*). This yield is higher compared to other polysaccharides extracted from other algae species: *Ulva lactuca* (11.7%) [27], *Enteromorpha compressa* (4.3%) [28], and *Caulerpa lentillifera* (5.4%) [29], but lower than *Ulva intestinalis* (17.9%) [30], *Caulerpa racemosa* (36.4%) [31], *Gracilaria lemaneiformis* (22.4%) [32], and *Ulva lactuca* (47.3%) [33]. The variability in the extraction yield could be due to different conditions, such as extraction parameters, ecological factors, physiological conditions, geographic location, growth cycle, and seasonal variations of seaweeds [34]. Furthermore, as illustrated in Table 1, PSHT contains many carbohydrates and small amount of uronic acid and protein.

Proteins are components of cell walls and are closely related to polysaccharides. They have been identified as a possible polysaccharide contamination [35]. Following depigmentation and PSHT extraction, we sought to denature protein and remove most fat. As a result, we obtained comparatively low protein levels (1.9 ± 0.2%) compared to other polysaccharide references. The protein level depends mainly on the method of extraction and deproteinization processes. According to Fleury et al. (1991) [36], precipitation of protein during extraction at 100 °C contributes to their indigestibility.

Moreover, as represented in Table 1, the total sugar rate was around 70% ± 0.3, which is higher than polysaccharide rate extracted from *Chaetomorpha linum* (52.5%) [37] and *Bryopsis plumose* (61.7%) [38]. Uronic acids and sulfated group contents represent 17.2% ± 0.0 and 3.6% ± 0.3, respectively.

### 2.2. Spectroscopic Analysis

#### 2.2.1. FT-IR Spectrometric Analysis

**Figure 1:**
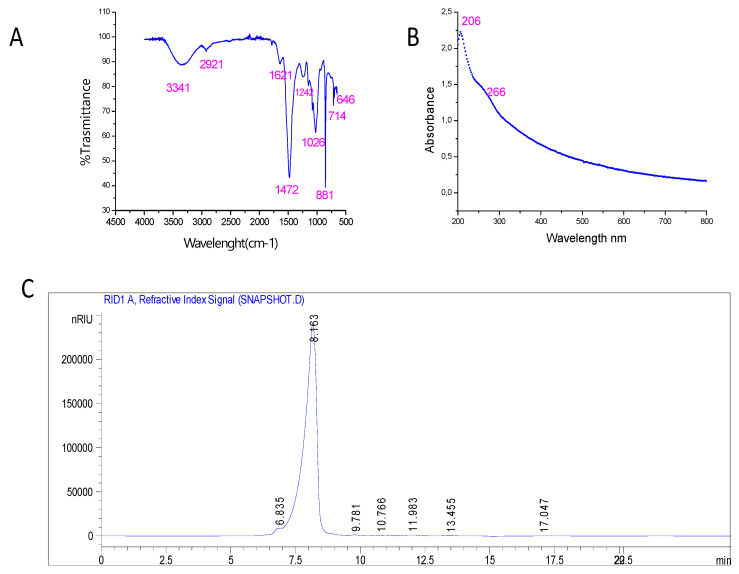
Preliminary characterization of PSHT. (**A**) Fourier transformed infrared spectrum, (**B**) UV–vis absorption spectrum, and (**C**) monosaccharide composition analysis by HPLC-FID.

FTIR spectrum obtained in the 4000–500 cm^−1^ region was used to determine the structure and glycosidic connections. As shown in Figure 1A, a prominent peak was detected at 3341, attributed to the stretching vibration of O-H. The absorbance peak at 2921 cm^−1^ is assigned to the C-H stretching vibration of polysaccharides. The signal detected at 1621–1472 cm^−1^ referred to the C=O trying vibration of the carboxylic group. Moreover, the peak at 1242 cm^−1^ suggested the presence of sulfated esters (S=O) [39]. The absorption band in the region 1026 to 1080 cm^−1^ indicates the presence of glycosidic band vibration (C-O-C). The band detected at 881 cm^−1^ is related and suggests the presence of 6- Sulfate galactose [39]. Finally, the absorbance peaks at the range 714–646 cm^−1^ are attributed to the α configuration of sugar. Based on the FT-IR spectrum of PSHT, it could be anticipated that the PSHT of *H. tuna* may be acidic and comprises galactose with an α-configuration.

#### 2.2.2. UV Absorption Peak Detection

The UV spectral analysis of PSHT (Figure 1B) revealed a significant peak at 206 nm, characteristic of polysaccharide, with a minor peak detected at 266 nm, indicating the presence of protein trace covalently attached to polysaccharide [40].

#### 2.2.3. Monosaccharide Analysis by HPLC-FID

The monosaccharide composition of PSHT was determined by HPLC-FID. As shown in Figure 1C, PSHT is mainly a hetero-polysaccharide with monosaccharide units, galactose, glucuronic acid, glucose, arabinose, xylose, fructose, and ribose at retention times 6.835, 8.136, 9.781, 10.766, 11.983, 13.455, and 17.047 min, respectively. Thus, data showed that the heterogeneity of PSHT with glucuronic acid was the most abundant compound. Previous studies performed on polysaccharides isolated from *Chaetomorpha linum*, green algae, also revealed heterogeneous compositions, including arabinose, mannose, and glucuronic acid, which were the major monosaccharide units of PS [37]. However, several studies reported that the chemical compositions and structures of polysaccharides from green seaweeds are very complex and vary from species to species. This variability in polysaccharide composition may be attributed to exogenous factors such as the temperature of extraction, the water concentration in nutrients, and extraction protocol, as well as endogenous changes in the organisms themselves, like the kind of species, growth, morphological changes, and reproduction cycle.

#### 2.2.4. X-ray Diffraction (XRD), Scanning Electron Microscopy (SEM)

**Figure 2:**
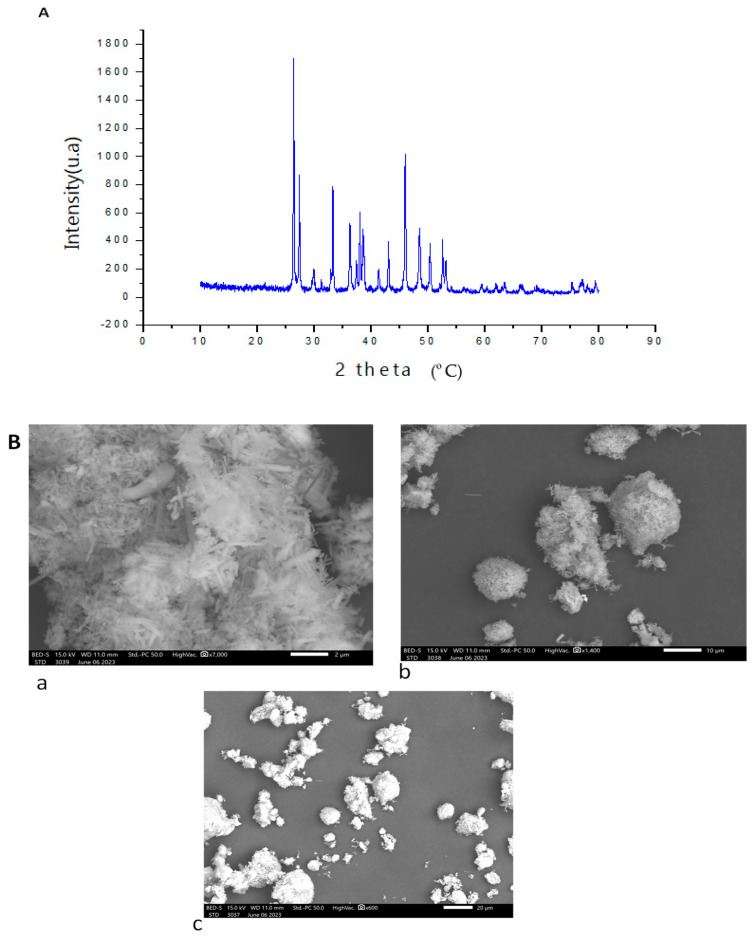
Structure characterization of PSHT. (**A**) X-ray diffraction pattern and (**B**) morphological examination of PSHT using scanning electron microscopy micrograph: (**a**)—2 µm, (**b**)—10 µm, and (**c**)—20 µm.

XRD was used to determine polysaccharides’ crystalline degree. As shown in Figure 2A, various peaks were detected, indicating a semi-crystalline structure of PSHT with four major crystalline reflections signaled at 26.41, 27.52, 33.41, 38.19, and 46.04°. Several studies found similar results with a semi-crystalline structure of polysaccharides [40]. Polymers’ crystalline and semi-crystalline structures are influenced by obvious physical properties, including tensile strength, flexibility, solubility, swelling, viscosity, or the opaqueness of the bulk polymer [41].

Scanning electron microscopy (SEM) evaluates biopolymers’ morphological structure. The microstructure of PSHT is presented in Figure 2B. It was observed that PSHT, an irregular and fragmented structure with many cavities, contributed to the water-holding capacity of polysaccharides. According to the literature, the bioactivity of the polymer is probably related to its structure. Contrary to our results, Gao et al. (2020) [42] demonstrated that polysaccharides from *Ulva pertusa* exhibited flakes with a smooth surface.

### 2.3. In Vitro Biological Properties of PSHT

#### 2.3.1. DPPH Free Radical Scavenging Assay

**Figure 3:**
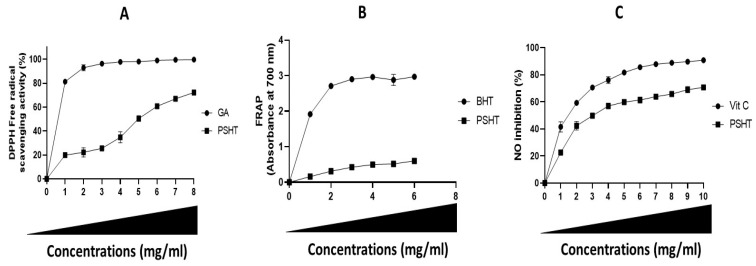
Anti-oxidant potentials of PSHT. (**A**) DPPH radical-scavenging activity, (**B**) reducing power capacity of PSHT, (**C**) nitric oxide (NO) inhibition.

Scavenging of DPPH radical activity is the test extensively used to determine the scavenging activity of natural polymers [40]. As shown in Figure 3A, data revealed a critical concentration-dependent anti-oxidant capacity of PSHT. A maximum DPPH inhibition was obtained at 5 mg/mL of PSHT. Several studies indicated that the anti-oxidant activity of polysaccharides is related to their monosaccharide composition, molecular weight, and configuration [37]. According to the literature, the sulfate content in polysaccharides significantly affects the radical scavenging capacity of polysaccharides [43]. Furthermore, the anti-oxidant capacity of sulfated polysaccharides was attributed to strong hydrogen donating ability [44].

#### 2.3.2. Reducing Power Assay

The reduction in the Fe^3+^/ferricyanide complex significantly indicates a potent anti-oxidant activity [45]. As shown in Figure 3B, the reducing power of PSHT and BHT (butylated hydroxytoluene) was monitored in a dose-dependent manner. At a 5 mg/mL concentration of PSHT, the maximum reducing power was around 0.599 (OD 700 nm). Still, this activity was lower compared to the standard anti-oxidant BHT (2.9 at OD 700 nm), according to Eljoudi et al. (2022) [46]. The reduced capacities of polysaccharides are generally due to bioactive compounds such as uronic acids, sulfates, and monosaccharides. Gunasekaran et al. (2021) [47] showed that the reducing power activity of polysaccharides was due to bioactive compounds such as uronic acid and monosaccharide composition. The polysaccharide extracted from *Pleurotus eous*, designated as P3a, exhibits an optical density (OD) of 0.9 at a concentration of 5 mg/mL. Following sulfation, the modified polysaccharide, referred to as SP3a, displays an OD of 1.1 at the same concentration of 5 mg/mL.

#### 2.3.3. Determination of Nitric Oxide Inhibition

Endothelial cells, neurons, and macrophages play a crucial role in many physiological and pathological processes, such as inflammation, through generating a vital chemical mediator, NO. This compound alters many cellular components’ structural and functional behavior. NO plays two roles. At lower quantities, it regulates physiological processes in the body; however, at greater concentrations, it is toxic, especially for bacteria or tumor and host cells [48].

The procedure of NO determination is based on the principle that, at physiological pH, sodium nitroprusside in an aqueous solution spontaneously generates NO, which interacts with oxygen to produce nitrite ions that can be estimated using the Griess reagent. Scavengers of NO compete with oxygen, leading to reduced production of nitrite ions.

Sulfate polysaccharide extracted from green algae *H. tuna* exhibited promising inhibition of NO production, with maximum activity at 1 mg/mL (70% ± 1.3). Vitamin C was used as a positive control. At 1 mg/mL, it induced 91 ± 0.2 NO inhibition (Figure 3C). Thus, the effect of PSHT in reducing NO levels could be related to its anti-oxidant activity. These findings are in line with those reported by Ben Saad et al. (2023) [49]. These authors claimed that polysaccharides extracted from red alga *Alsidium corallinum* exhibited a maximum NO scavenging activity at 1 mg/mL with inhibition of 40%.

According to El-Sheekh et al. (2023) [50], fucoidan from the brown alga *Dictyota dichotoma* (at different levels in sodium nitroprusside solution) could decrease the nitrite levels with an IC_50_ of 9.3 mg/mL by their ability to chelate nitric oxide.

These findings align with those found by Ananthi et al. (2010) [51]. These authors showed that water-soluble crude polysaccharides obtained from the brown alga *Turbinaria ornata* exert a maximum scavenging activity at 125 µg/mL with an inhibition of 38.8%.

#### 2.3.4. Anti-Hemolytic Activity and the Impact of PSHT in Stabilizing Erythrocyte Membranes In Vitro

Membrane stabilization has been used to investigate the in vitro anti-inflammatory activity since the erythrocyte membrane is analogous to the lysosomal membrane. Lysosomal stabilization is essential in limiting the inflammatory response. It prevents the release of lysosomal constituents of activated neutrophils, such as bacterial enzymes and proteases, which cause further tissue inflammation and damage. The lysosomal enzymes released during acute or chronic inflammation induce various disorders [46]. Some drugs’ effect on the erythrocyte membrane stabilization could indicate lysosomal membrane stabilization that modulates the release and/or the action of some mediators like histamine, serotonin, prostaglandins, and leukotrienes [52].

**Figure 4:**
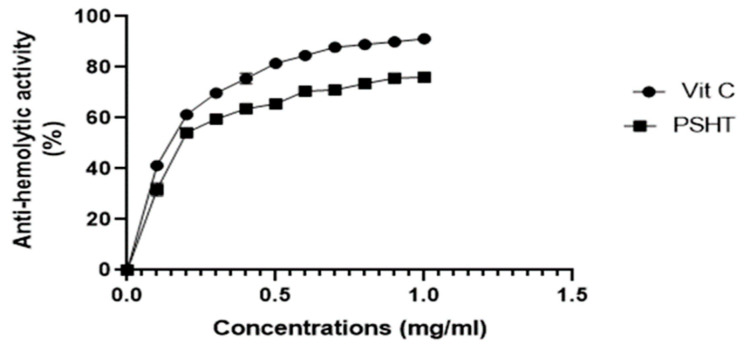
Anti-hemolytic activity of PSHT in vitro.

This is the first study evaluating the anti-hemolytic activity of a crude polysaccharide extract from green seaweed *H. tuna* and its impact on blood erythrocytes membrane from healthy donors exposed to hypotonic-induced lysis in vitro. Vitamin C was used as a standard control drug (Figure 4). At a 1 mg/mL concentration, PSHT and vitamin C inhibited the erythrocyte hemolysis by 76% and 91%, respectively. According to previous research of Fideled et al. (2022) [53], the aqueous extract of *Garcinia kola* (Clusiaceae), fresh seeds named EAgk, exhibited anti-oxidant activity, thereby protecting erythrocytes from hemolysis. The percentage of hemolysis inhibition of EAgk was increased in a concentration-dependent manner, with an IC_50_ value of 1.0 ± 0.2 mg/mL compared with 0.6 × 10^−2^ ± 0.5 mg/mL for ascorbic acid, which served as positive control.

Sulfated polysaccharide extracted from *Navicula incerta* registered 90% hemolysis inhibition, achieving integrity of the erythrocyte membrane [54]. During inflammation, lysosomal enzymes and hydrolytic components are released from the phagocytes to the extracellular space, which causes damage to the surrounding organelles and tissues and also leads to several disorders [50,51].

Based on our results, the sulfated polysaccharide extracted from *H. tuna* could stabilize the red blood cell membrane against hypotonic solution and heat-induced lysis. Therefore, the PSHT could be considered as a natural source of membrane stabilizers and could be used as an alternative remedy for the management and treatment of inflammatory-related disorders and diseases.

Previous studies have demonstrated that polysaccharides from vegetal sources exhibited anti-hemolytic properties [52]. Those with sulfate groups are endowed with more important anti-hemolytic activity [55]. For example, Wang et al. (2008) [56] extracted three components from *Laminaria japonica*, F1, F2, and F3, with sulfate contents of 23.3%, 36.4%, and 36.7%, respectively, and their ability to scavenge superoxide anion, hydroxyl radical, chelating ability, and reducing power all decreased in the order of F3 > F2 > F1, suggesting that sulfate content was positively related with the bioactivities of a polysaccharide. Overall, due to their excellent biocompatibility and biodegradability, sulfate polysaccharides are suitable candidates for pharmaceutical and biotechnology applications [57].

## 3. Effect of PSHT in RAW264.7

### 3.1. Cell Viability Assay

Macrophages play critical roles in innate immune response and host defense [1] regarding their potential to secrete several effectors and immune mediators and their ability in recognizing and eliminating pathogens and damaged cells. In fact, upon exposure to inflammatory stimuli such as lipopolysaccharides (LPS)-induced activation of macrophages involves the TLR4 and results in the production of pro-inflammatory cytokines like TNFα, IL1β, IL6, IL8, IL10, IL12, IL15, IL-18, and TGFβ, and the production of inflammatory mediators such as NO, PGE2, and ROS.

Based on their response to different stimuli by releasing NO and immune-related cytokines in vitro [55,58,59], the murine macrophage RAW264.7 cells are frequently used as a model for in vitro evaluation of the immune regulatory activity of several compounds. As showed by Aki et al. (2020) [60], LPS stimulated RAW264.7 cells and induced the production of cytokines and the production of inflammatory mediators.

The evaluation of the viability of RAW 264.7 murine macrophages has previously been used as an indicator of the activation of macrophages and, subsequently, for the characterization of the immunomodulatory potential of new compounds with biomedical applications [61].

**Figure 5:**
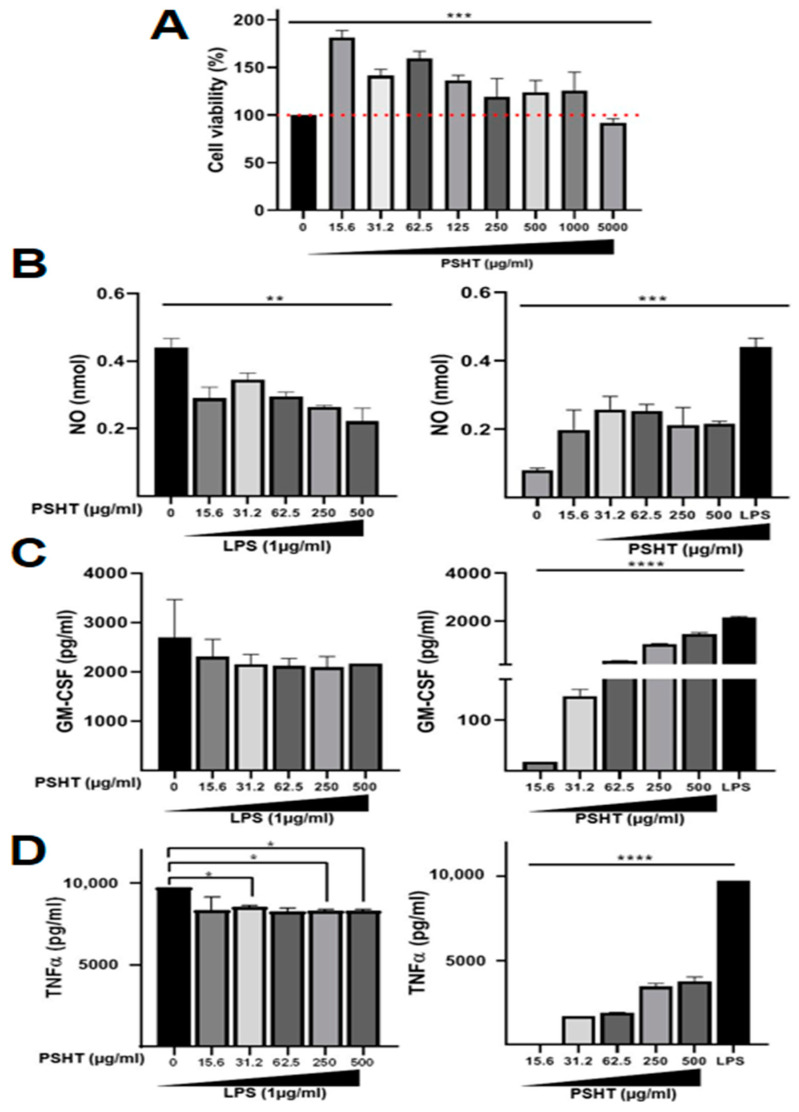
Effect of PSHT on RAW 264.7 macrophages. (**A**) Protective effects of PSHT extracts on the viability of RAW 264.7 macrophages measured by the MTT assay. Cells were pretreated with vehicle or with different concentrations of PSHT. (**B**) Effect of PSHT on NO release into the medium by LPS-activated RAW 264.7 macrophages. A Griess reagent determined the nitrite content in the medium. (**C**) Effect of PSHT on GM-CSF production by LPS-activated RAW 264.7 macrophages. (**D**) Effect of PSHT TNF-α secretion by LPS-activated RAW 264.7 macrophages. Each value is the mean ± SD (*n* = 3). * Comparison of all groups versus control group * *p* < 0.05, ** *p* < 0.01 and *** *p* < 0.001, **** *p* < 0.0001. Comparison of PSHT and LPS treated groups versus LPS treatment.

Therefore, in this study, we first investigated the effect of different concentrations of PSHT (15.6–5000 μg/mL) on the viability of RAW 264.7 macrophages after 24 h of treatment. PSHT did not exhibit cytotoxic activity on macrophages (Figure 5A). Interestingly, this extract significantly increased the viability of RAW 264.7 cells, reaching 181% at a concentration of 15.6 µg/mL (Figure 5A). Thus, compounds activating macrophages promise to improve the host immune system ability [62,63,64,65]. 

A previous study reported that natural polysaccharides (LDP-1) extracted from the fruiting bodies of a rare wild *Lactarius delicious* with a molecular weight of 9.8 × 10^5^ Da are non-toxic and exhibited immunological activity in the RAW 264.7 cells [63]. Another study [64] indicated that sulfate polysaccharide from green seaweed *Ulva lactuca* (ULPF) had no apparent toxicity toward RAW264.7 proliferations at 10–60 μg/mL. However, at 60 μg/mL, ULPF exhibited a weak cytotoxic activity on murine RAW264.7 macrophages. The differences in RAW264.7 cell cytotoxicity were probably related to the differences in the complex structures of sulfate polysaccharide, including monosaccharide composition, molecular weight, degree of branching, the presence of sulfate groups, and glycosidic linkages [59]. For example, Jian et al. (2022) [65] extracted three kinds of polysaccharide (PEPs) with different molecular weights (Mws): high-Mw PEP (HPEP), medium-Mw PEP (MPEP), and low-Mw PEP (LPEP) from *Enteromorpha prolifera.* The authors showed that hydrolyzed low-molecular-weight polysaccharide exhibited a higher anti-inflammatory activity. However, Liu et al. (2022) [66] demonstrated that the polysaccharide SCP-1-1 extracted from *Sinonovacula constricta* had no cytotoxic effect on RAW264.7 cells at concentrations ranging from 300 to 1500 μg/mL.

### 3.2. Determination of Nitrite Production

NO is one of the most inflammatory mediators involved in host defense against pathogens [67]. Activated macrophages could synthesize NO, an essential cytotoxic/cytostatic mechanism of non-specific immunity [1]. PSHT reduced NO levels in LPS-stimulated RAW 264.7 macrophages, confirming its immunomodulatory and anti-inflammatory effects (Figure 5B). The sulfated polysaccharide from *H. tuna* decreased the NO levels in LPS-treated macrophages, consequently regulating the macrophage’s inflammatory response in vitro. The NO production in RAW264.7 could be related to the upregulation of iNOS by PSHT. It has been reported that the polysaccharide ESPS-CP from *Padina tetrachromatic* algae and the sulfated polysaccharide from *Codium fragile* modulated NO and iNOS production in RAW264.7 cells [63]. Similarly, Mezdour et al. (2017) [64] demonstrated that ULFP from *Ulva lactuca* induced a potent NO inhibition of LPS-stimulated RAW264.7 cells (71.4% at 60 μg/mL).

### 3.3. PSHT Effect on GM-CSF and TNF-α Production In Vitro

The inflammatory response is coordinated by a panel of regulators and effectors [68]. Indeed, macrophage activation by lipopolysaccharide (LPS) leads to the secretion of several pro-inflammatory cytokines such as GM-CSF, TNF-α, interleukin-6 (IL-6), and interleukin-8 (IL-8) [68], that play a pivotal role in the ordinarily protective immune response [61]. However, the exacerbated production of such mediators contributes to the development of chronic inflammation and related diseases [69].

Interestingly, we found that PSHT exhibited both immunomodulatory and anti-inflammatory effects in RAW 264.7 macrophages. As shown in Figure 5C,D, compared to mock-treated cells, PSHT significantly enhanced the production of GM-CSF and TNF-*α* in RAW 264.7 macrophages, which is concordant with its effect on cell viability and NO production. Interestingly, in response to LPS stimulation, PSHT partially decreased GM-CSF and TNF-α secretion in RAW 264.7 macrophages. Our results are in agreement with those of Peng et al. (2018) [70] who reported that fucoidan extracted from *Kjellmaniella crassifolia* and *Undaria pinnatifida* marine algae exhibited immunomodulatory activity by stimulating macrophage cell proliferation and increasing the secretion of various cytokines such as GM-CSF and TNF-α. These data are in line with the study of Mezdour et al. (2017) [64] reporting that sulfate polysaccharide extracted from the green algae *Ulva lactuca* exerted a significant dose-dependent capacity to down-regulate macrophage production of NO, TNF-α release, and exhibited an anti-inflammatory effect [64].

A thorough understanding of PSHT immunomodulatory effects will facilitate more appropriate use and thus further enhance its anti-inflammatory activity [64].

## 4. In Vivo PSHT Application

In the current work, we asked whether PSHT’s in vitro anti-inflammatory effect in RAW 264.7 macrophages could be extended to the in vivo setting. For this reason, carrageenan-induced edema in the rat paw model was used to investigate the physiopathology of acute local inflammation and to test the potential anti-inflammatory effect of PSHT as a new molecule. Thus, we investigated the impact of the sulfated polysaccharide PSHT on experimental CAR-induced acute inflammation using biochemical and histological tools.

### 4.1. Effect of PSHT in Carrageenan-Induced Paw Edema

**Figure 6:**
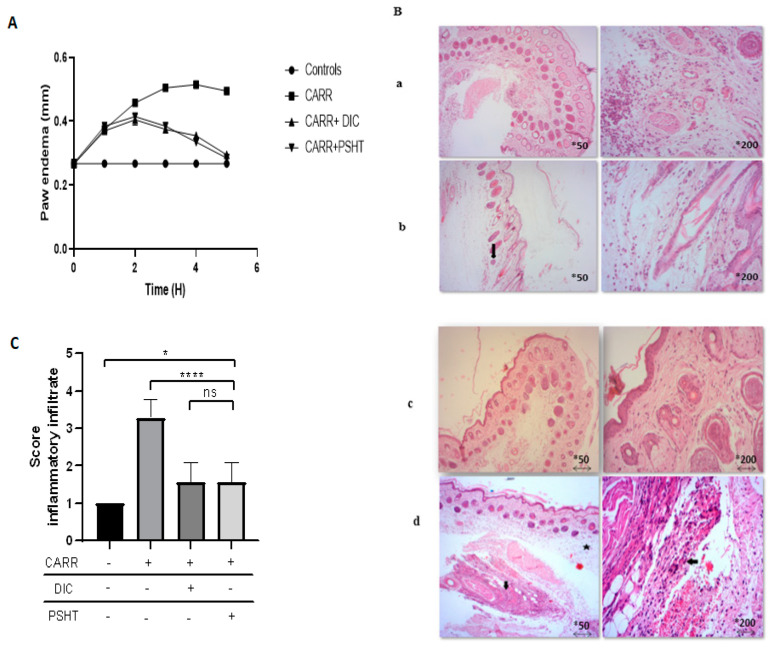
Effect of PSHT on induced inflammation in vivo: (**A**) Effect of PSHT and diclofenac (DIC) on carrageenan-induced paws edema. (**B**) Representative skin photographs confirmed PSHT’s protective effect against carrageenan-induced inflammation (H&E-stained skin sections examined with a light microscope magnification ×50 (scale bar = 100 µm) and ×200 (scale bar = 50 µm). (**C**) Semi-quantitative scores of inflammatory infiltrates in the histological observation of adult rats. Controls: (**a**) rats treated with NaCl 0.9%; CARR (**b**): rats treated with carrageenan 1%; CARR + DICL (**c**): rats injected with CARR and gavaged with DIC 20 mg/kg; CARR + PSHT (**d**): rats injected with CARR and gavaged with PSHT 20 mg/kg. Values are expressed as means ± SD for six animals in each group. * Comparison of all groups versus control group * *p* <0.05, **** *p*< 0.0001, ns: not significant. Comparison of PSHT and DIC treated groups versus CARR treatment →: lymphocytic infiltration. ★: Edema.

The role of sulfate polysaccharide extracted from green seaweed *H. tuna* on carrageenan-induced acute inflammation model was evaluated at a 20 mg/kg concentration. As shown in Figure 6A, the evolution of the edema induced severe swelling 3 h after the administration of CARR and was maintained until 5 h. The inflammatory response of CARR administration consisted of two phases. The first (1–3 h) was marked by swelling developed due to enhanced vascular permeability and neutrophil recruitment from vessels to the damaged area. It is mediated by serotonin and histamine. The second phase (3–5 h after administration) was characterized by the production of prostaglandins. The CARR administrated to animals induced an increase in the size of edema compared to the control group. However, a significant decrease was noted in the CARR + PSHT and CARR + DIC groups compared to the CARR group. Our results highlighted a significant reduction in edema size in PSHT and DIC-treated groups compared to the control group registered from the third hour after an injection of CARR. The decrease in the size of edema in rats receiving PSHT was similar to those treated by DIC used as positive controls during the entire period of the experiment. Previous data regarding CARR injection (1%) in rat hind paw induced progressive swelling of the rat paw with a maximum level at the third hour (6.8 ± 0.3 mm) and reported that the sulfated polysaccharide displayed a significant reduction in the size of edema [64]. The percentage of edema inhibition by diclofenac and PSHT with 100 µL of carrageenan (1%) was summarized in Table 2. Co-treatment with PSHT (20 mg/kg) showed a significant decrease in edema after 5 h of carrageenan administration. When the PSHT was administered orally, the percentage of inhibition reached 48.6% after 3 h. At the fifth hour, PSHT exhibited maximum edema inhibition (88.7%), like diclofenac which induced 88.9% edema inhibition. In this way, sulfated polysaccharides extracted from green seaweed *Ulva lactuca*, named ULPF, displayed a significant anti-edema activity after 3 h of treatment. This effect is clearly compared to diclofenac–sodium (a standard drug) [64].

In the current study, sulfate polysaccharide extracted from *H. tuna* showed a promising anti-inflammatory activity at 20 mg/kg. This is marked by the ability to reduce the effect of carrageenan injection in the rat paw, representing a well-established model of inflammation used in non-steroidal, anti-inflammatory drug development. However, the inflammatory response is usually quantified by swelling and edema after carrageenan administration, and it is modulated by specific inhibitors in the phase of carrageenan-induced edema via inhibition of mediators such as histamine and NO.

The histopathological severity of the inflammatory response in paw tissues specimens from different groups was scored according to the microscopic examination of the skin specimens. As shown in Figure 6B, sections were examined from rats treated with CARR, PSHT, DIC, and an untreated group for 5 h before CARR injections. The standard saline-treated group showed the presence of typical histological structure of both the epidermis and dermis. Inflamed groups with CARR showed the predominance of inflammatory cells in the deep dermis associated with vascular congestion (hyperemia) and dilated capillaries. However, the DIC and PSHT skin specimens showed histological changes by decreasing the inflammatory cells in the dermis and inside the blood vessel. These data were confirmed by a histological score calculated for each section (Figure 6C). This finding is in agreement with the study of Sousa et al. (2018) [71] showing that polysaccharide extracted from *Morindacitrifolia Linn* (Noni) decreased the microscopic inflammatory reaction in paw tissue caused by carrageenan at 10 mg/kg doses.

The potential effect of PSHT on reducing the damage caused by CARR could be due to its ability to modulate macrophages response (Figure 5A–D). Macrophages and polynuclear play an essential role in the immune system; they are vital members of the host’s natural immune defense [72]. Activated macrophages can kill pathogenic microorganisms through phagocytosis by secreting and regulating NO and other immunologically active molecules involved in the evolution of the immune reaction [1]. Our findings are in line with those of Mezdour et al. (2017) [64], who showed that sulfate polysaccharide extracted from *Ulva lactuca* displayed a significantly regulated NO production and TNF-α release in RAW264.7 and exhibited significant inhibition of carrageenan-induced-damages.

The CARR causes overproduction of ROS at the site of inflammation and cell induces tissue damage leading to several health disorders, including cancer and inflammatory diseases. Thus, it is necessary to study the cellular state of the pro/anti-oxidant system. Indeed, an imbalance in the redox state can induce the occurrence of severe cellular complications, going until cell death.

**Figure 7:**
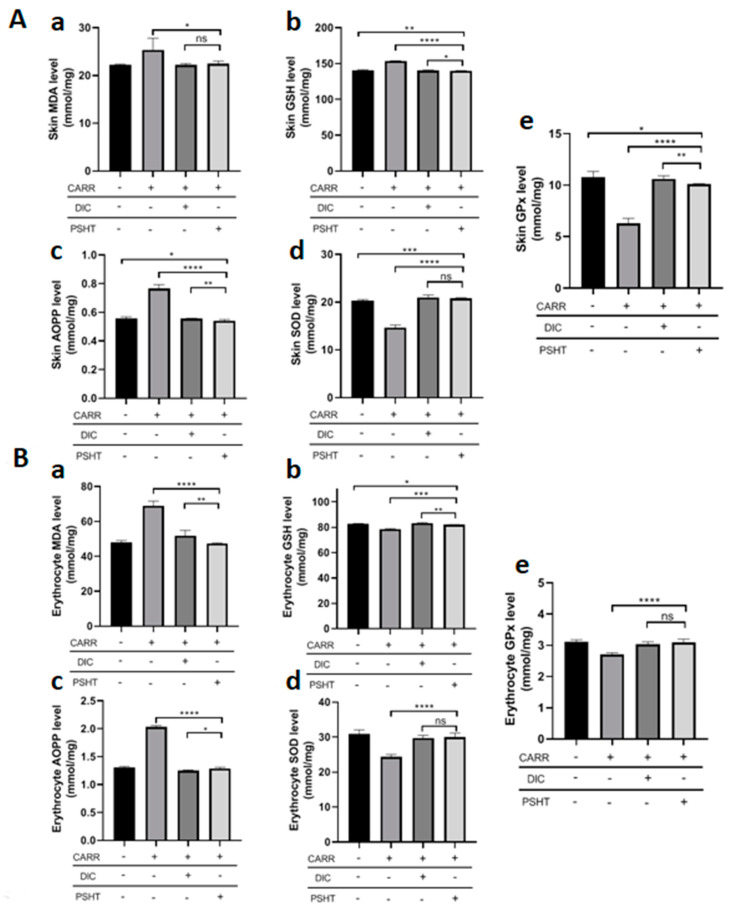
Effects of Carrageenan, PSHT and DICL on the levels of MDA and AOPP and on GPx, SOD, and GSH activities in skin (**A**) and in erythrocytes (**B**). (**a**) Evaluation of MDA levels. (**b**) Evaluation of GSH levels. (**c**) Evaluation of AOPP levels. (**d**) Evaluation of SOD activity. (**e**) Evaluation of GPx activity. Controls: rats treated with NaCl 0.9%; CARR: rats treated with carrageenan 1%; CARR + DICL: rats injected with CARR and gavaged with DIC 20 mg/kg; CARR + PSHT: rats injected with CARR and gavaged with PSHT 20 mg/kg. Values are expressed as means ± SD for 6 animals in each group. * *p* < 0.05, ** *p* < 0.01, *** *p* < 0.001, **** *p* < 0.0001, ns: not significant.

The AOPPs are indicators of protein oxidation due to the action of free radicals generated by activated neutrophils and involved in inflammation. Additionally, MDAs are commonly known as a marker of lipid peroxidation and oxidative stress cell installation. Our experiments showed that the pro-oxidant levels increased 5 h after carrageenan injection by inducing progressive production of MDA and AOPP levels of the inflamed group in skin compared to healthy rats, as seen in Figure 7. Nevertheless, DIC treatment and PSHT alone significantly decreased the AOPP and MDA levels. To explore the effects of PSHT on anti-oxidant defense, the anti-oxidant activities of GPx, GSH, and SOD were carried out (Figure 7).

SODs are metalloproteins that catalyze the conversion of superoxide anion to oxygen (O_2_) and hydrogen peroxide. CARR injection significantly reduced SOD levels (−27.6%) in comparison to the untreated group. In contrast to CAR-injected paws, treatment with PSHT and DIC restored these changes to reach normalcy and improved oxidative alterations.

Glutathione peroxidase (GPx) is essential for reducing hydroperoxides and preventing lipid and protein oxidation. GPx activity is important in regulating oxidative stress in the cell. Compared to the control, CARR injection resulted in a significant decrease in GPx activities. However, PSHT treatment increased in GPx activity. The GPx activity in all samples is similar in the PSHT and DIC groups.

Glutathione (GSH) protects cells from oxidative damage and the toxicity of xenobiotic electrophiles and maintains redox homeostasis. After 5 h of CARR injection, the levels of GSH showed a significant decrease (*p* < 0.001) compared with the untreated group. However, in animals treated with PSHT and DIC, the GSH decreased remarkably (−8.9% and −8.5%, respectively). Our results showed that PSHT restore these changes to reach normalcy which could be explained by the promising anti-oxidant activity of sulfate polysaccharides.

### 4.2. Effect of PSHT on Hematological Parameters

Subplantar injection of CARR produced significant changes in hematological parameters (WBC, PLT, RBC, LYM, and Hb) in all the groups after 5 h of the administration of CARR. However, MCH, MCHC, and HCT remained unchanged.

As shown in Table 3, compared with the healthy group, we observed a highly significant increase in WBC, PLT, LYM, and RBC. However, treatment of rats with CARR improved the reduction in RBC compared to control groups. Our experimentation showed that CARR administration decreased RBC count (*p* ≤ 0.05) compared to the control group. Similar to our results, the study of Zammel et al. (2017) [73] showed that RBC count decreased in the CARR group.

During inflammation, the production of several enzymes was increased, such as mainly nitric oxide synthase and reactive oxygen species, which can lead to tissue damage. It is known that ROS influences RBCs’ deformability and degradation during inflammation. In the current study, MDA and AOPP levels were evaluated in erythrocytes. After 5 h of CARR injection, the levels of MDA and AOPP were remarkably decreased compared with controls (+44.0 and +55.8%, respectively, *p* < 0.001). In groups treated with PSHT and DIC, the levels of MDA and AOPP demonstrated a remarkable decrease. The co-administration of DIC and PSHT increases the levels of SOD and GPx (Figure 7). These results indicated that sulfate polysaccharide from *H. tuna* can protect erythrocytes against inflammation-induced lipid and protein peroxidation. This effect could be related to the promising anti-oxidant activity of sulfate polysaccharide to minimize ROS damage, hemolytic activity, and immune system disorders.

The inflammatory response consists of increased blood vessel permeability, leading to the migration and activation of PMNs. The activation of immunocytes, such as monocytes and macrophages, manifests in inflammation. An inflammatory condition involves the induction of edema and the recruitment of cells, predominantly neutrophils, to the site of inflammation. In addition, a significant rise in WBC and PLT occurred in the 5th hour following the CARR injection. However, treating rats with PSHT and DICL significantly improved the WBC and PLT counts (*p* ≤ 0.05; *p* ≤ 0.01, respectively) compared to the CARR group. In addition, no significant difference was shown between the PSHT and DICL groups.

**Figure 8:**
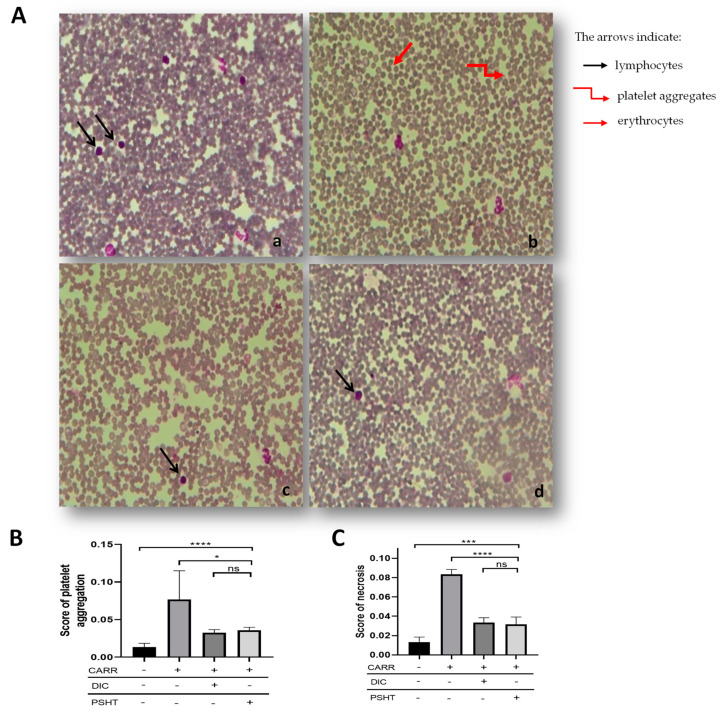
Effect of PSHT on blood smears: (**A**) blood smears stained with May–Grunewald–Giemsa examined with a light microscope by ×400 magnifications (scale bar = 50 µm). (**B**,**C**) Semi-quantitative scores of platelet aggregates and necrosis in the blood smears of adult rats. Controls (**a**): rats treated with NaCl 0.9%; CARR (**b**): rats treated with carrageenan 1%; CARR + DICL (**c**): rats injected with CARR and gavaged with DIC 20 mg/kg; CARR + PSHT (**d**): rats injected with CARR and gavaged with PSHT 20 mg/kg. Values are expressed as means ± SD for 6 animals in each group. * *p* < 0.05, *** *p* < 0.001, **** *p* < 0.0001 and ns: not significant.

The results of the blood smear confirmed the hematological results (Figure 8A–C and Table 4). In the inflamed group, the blood smear showed an increase in lymphocytes (+48%) and neutrophils (+50%); conversely, co-administration with PSHT and DIC improved these parameters compared to the healthy group. Our results align with previous reports showing that sulfated polysaccharide regulated WBC counts in active inflammation [58].

### 4.3. Effect of PSHT on Inflammation-Related Serum Protein Levels

Acute phase reactants (APR) are inflammation markers. Their serum concentrations change significantly during inflammation. These are also important mediators that the liver produces during acute and chronic inflammatory states [74]. The liver is the primary site of serum protein synthesis, such as albumin and globulin, which is stimulated by the release of interleukins and cytokines during localized inflammation and plays a role in inflammatory response regulation. According to previous research by Varela et al. (2018) [75], APPs can detect inflammatory and infectious disorders.

Albumin, the main serum protein, reflects the nutritional status and the acute-phase response to inflammation, and its levels decrease following the inflammatory stimulus. Globulin, another abundant serum protein, is required for immunity and inflammation.

Based on our results, it is speculated that the injection of CARR significantly decreases the albumin level, which reached about 18 ± 0.3 g/dL, compared with the control group (22.1 ± 0.4 g/dL). This result aligns with Kaysen et al. (2005) [76], who reported that albumin is also a harmful acute-phase protein with levels that decrease during the inflammatory response.

As shown in Table 5, injection of CARR into the rat’s paw significantly increased total protein levels in the blood (*p* < 0.001) by 16.4% compared to the inflamed group. Protein concentrations of Alpha1-globulin, Beta1-globulin, and Gamma-globulin were also significantly higher in the inflamed group by 52.3%, 12.5%, and 8.2%, respectively. Treating rats with PSHT and DICL significantly decreased the levels of total proteins, Alpha1, Beta1, and Gamma globulin protein, which were almost identical to the healthy group. This observation can be attributed to various sources and is consistent with CARR’s harmful effects on the immune system and liver cells. Acute phase proteins are essential in the inflammatory response at multiple stages. According to the study of Sproston and Ashworth. (2018) [77], the impacts of chemical mediators of inflammatory reactions might explain all protein profile changes. Interestingly, PSHT lowered these inflammatory markers that had increased following the inflammatory response. The alleviative effect is undoubtedly connected to PSHT’s ability to regulate APP synthesis in the liver, reducing immune cell activation, and creating pro-inflammatory mediators. This indicates that the tested polysaccharide has potential inhibitory effects on acute inflammation.

## 5. In Silico Findings

Considered as a significant regulator of inflammation and involved in the pathogenesis of both immune and inflammatory diseases [45,69,73], the interaction between COX-2 and TNF-α when complexed to the monosaccharides has been analyzed to understand the molecular interactions.

The computational analyses showed that the monosaccharides resulted in stable complexes with COX-2 and TNF-α. All binding energies were negative and reached −6.6 Kcal/mol. Considered a significant regulator of inflammation and involved in the pathogenesis of both immune and inflammatory diseases [78,79], the interaction between COX-2 and TNF-α when complexed to the monosaccharides has been analyzed to understand the molecular interactions better. Complexes exhibiting good stability, indicated by a root-mean-square deviation (RMSD) equal to 0.0 nm, were analyzed following previously reported procedures [80,81].

Attractive and conventional H-bonds are important in biological activities, including anti-oxidant, anti-hemolytic, and anti-inflammatory effects [80,82]. The studied monosaccharides established 4 to 7 or 8 conventional H-bonds with COX-2 and TNF-α, respectively (Table 6).

**Figure 9:**
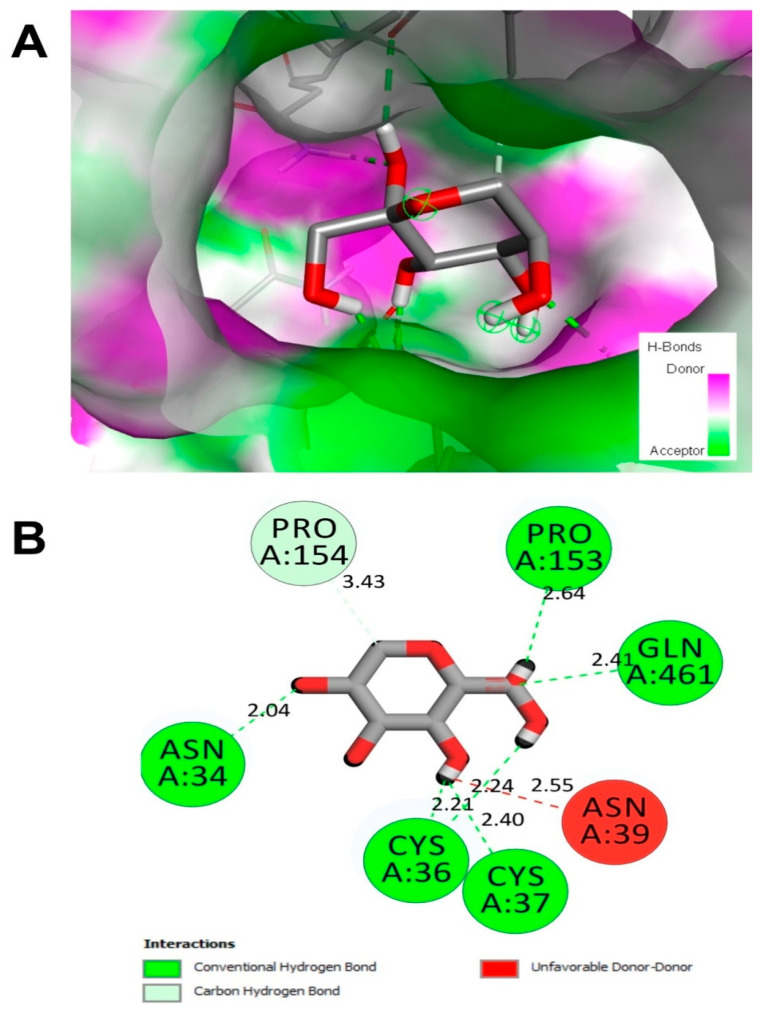
(**A**) H-bond tridimensional illustrations of D-fructose bound to COX-2 receptor exhibit the best binding affinity to get her with the highest number of closest interacting residues. (**B**) The corresponding diagram of interactions. Note the six conventional H-bonds and the involvement of 7 different amino acids, including Asn34, which was deeply embedded.

These interactions involved 3 to 7 amino acid residues near the target receptors. All distances estimated between the complexed ligands and receptors were less than 2.5 Å. In this context, the deepest embedded monosaccharide was d-galactose while docked to TNF-α, in which Asn46 exhibited a distance of 1.85 Å only. D-fructose was chemically interacting with Asn34 (2.04), Pro153 (2.64), Gln461 (2.41), Asn39 (2.55), Cys37 (2.40), and Cys36 (2.21) (Figure 9). Deeply embedded, H-bonds, and chemical interaction types are commonly assessed as they are well involved in pharmaceutical approaches and drug design [49,80,83]. These in silico findings may explain the current experimental results and support the previously published reports about the beneficial effects of *H. tuna* products.

## 6. Materials and Methods

### 6.1. Algal Material

**Figure 10:**
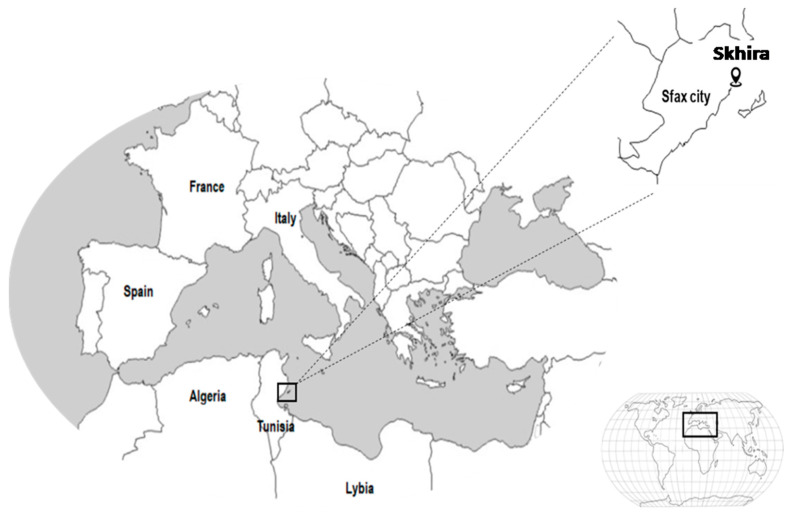
Location of the collection zone (the coastal area of Skhira, Sfax, Tunisia).

Green seaweed *Halimeda tuna* was collected from the coastal area Skhira, Sfax, Tunisia (Sfax, Tunisia; 34°17′ N, 10°04′ E—Figure 10) in March 2020, and the voucher sample was deposited at the laboratory. It was washed with distilled water and dried at room temperature for 15 days. The drained algae were powdered in the grinder and preserved in a limp sterile for further studies.

### 6.2. Extraction Yield and Chemical Characterization

The polysaccharide extraction procedure was performed according to the method described by Feki et al. (2019) [84], using hot water for extraction and ethanol as precipitating agents. The seaweed flour (50 g) was dispersed in distilled water, stirred at 90 °C for four hours, and filtered. The filtrate was centrifuged at 3600× *g* for 10 min. After centrifugation and concentration, the ethanol was added (one-third volume) to a concentration of 95% for alcohol precipitation at 4 °C for 24 h. The PSHT was obtained after centrifugation using a refrigerated centrifuge and lyophilization. The yield was calculated as the percentage ratio of the dry weight of the extracted polysaccharide (in grams) to the dry weight of *H. tuna* (in grams).

Carbohydrate content was quantified by the phenol sulfate acid method [37].

Soluble proteins in PSHT were determined by referring to a colorimetric assay [85]. The carbazole–sulfate method [86] evaluated the content of the uronic acid. Sulfate content was determined according to the gelatin–barium method [87] using potassium sulfate (K_2_SO_4_) as standard (1 mg/mL).

### 6.3. Spectroscopic Analysis

#### 6.3.1. FT-IR Spectrometric Analysis

Infrared (IR) spectrum analysis of PSHT was determined at room temperature using a Nicolet Nexus spectrometer; the spectrum was recorded in the wave numbers range of 4000–500 cm^−1^. OPUS.3.0 data gathering software was used to evaluate the spectral data (Bruker, Ettlingen, Germany).

#### 6.3.2. UV Absorption Peak Detection

The UV spectrum of PSHT was determined in the wavelength range of 200–800 nm.

#### 6.3.3. X-ray Diffraction (XRD)

X-ray Diffraction (XRD) patterns of PSHT were determined at room temperature on an X-ray diffractometer (D8 advance, Bruker, Germany). Data were gathered within the 2θ range of 5–8, employing a step size of 0.05° and a counting time of 5 s.

#### 6.3.4. Monosaccharide Analysis by HPLC-FID

The HPLC-FID assay was performed according to Eljoudi et al. (2022) [46]. The PSHT (2 mg) was dissolved in 4 mol/L TFA at 100 °C for 2 h. Then, the TFA was removed by washing it with methanol. A total of 50 mg of sodium tetrahydruroborate was added to reduce the hydrolyzed product, then pyridine and acetic acid anhydride were added at 40 °C for 2 h to acetylation. The acetylated sample was filtered and analyzed by HPLC-FID using an Aminex HPX-87H column (Bio-Rad Laboratories, Hercules, CA, USA) with (H_2_SO_4_) as the mobile phase of (0.001 N), a flow rate of 0.4 mL/min, and a column temperature of 60 °C.

#### 6.3.5. Scanning Electron Microscopy (SEM)

The microstructure of PSHT was estimated by SEM (Termoscientific 250 microscope, SUPRA 55VP, Zeiss, Germany) operating at 3.0 KV. The sample was photographed with an angle 90° to the surface.

### 6.4. In Vitro Biological Properties of PSHT

#### 6.4.1. DPPH Free Radical Scavenging Assay

The DPPH radical scavenging capacity was estimated by Lopes-Lutz et al. (2008) [88]. The absorbance was determined at 517 nm with a spectrometer. The percent of inhibition (PI) was calculated using the equation below:

$$PI\left(\%\right)=\frac{Ac+Ab-As}{Ac}\times 100$$

where Ab, Ac, and As refer to the blank, control, and sample optical densities, respectively. All experiments were performed in triplicate with BHA as the positive control.

#### 6.4.2. Reducing Power Assay

The ability of PSHT to reduce iron (III) was evaluated according to the method of Oyaizu et al. (1986) [89]. The assay is based on the ability of an anti-oxidant molecule to reduce ferric iron from K_3_Fe (CN)_6_ to ferrous iron (Fe^2+^). Briefly, a 0.5 mL sample at different concentrations (0.25–5 mg/mL) was mixed with 1.25 mL of potassium phosphate buffer (0.2 mol/L; pH 6.6) and 1.25 mL of potassium ferricyanide (1% *w*/*v*). The mixture was then incubated at 50 °C for 30 min. After incubation, 0.5 mL of 10% (*w*/*v*) trichloroacetic acid (TCA) was added, and the reaction mixtures were then centrifuged for 10 min at 2000 rpm. Thus, 1.25 mL of each sample mixture was mixed with 1.25 mL of distilled water and 0.25 mL of 0.1% (*w*/*v*) ferric solution. The absorbance of the mixture was recorded at 700 nm. Higher absorbance of the resulting solution indicated higher reducing power capacity. The commercial anti-oxidant BHT (butylated hydroxytoluene) was used as standard. The experiment was carried out in triplicate, and the results were presented as mean values.

#### 6.4.3. Determination of Nitric Oxide Inhibition

The nitric oxide inhibition induced by PSHT was calculated using the method decrypted by Marcocci et al. (1994) [90]. The absorbance was measured at 546 nm, and the percentage of NO radical inhibition was calculated below:

$$\%\;NO\;inhibition=\frac{A0-A1}{A0}\times 100$$

A0 and A1 were the absorbance of the blank and polysaccharide solution, respectively.

### 6.5. Hemolytic Activity of Sulfate Polysaccharide

The hemolytic activity of PSHT was evaluated on erythrocyte suspension, according to Uddin et al. (2015) [91]. Briefly, fresh blood (10 mL) was collected from a healthy volunteer and transferred to heparinized tubes. They were centrifuged at 2500× *g* for 30 min and washed three times with an equal volume of normal saline. The blood volume was measured and reconstituted as a 10% *v*/*v* suspension with normal saline.

The reaction mixture (2 mL) consisted of 1 mL of PSHT extracts at concentrations ranging from 0.5 to 2.5 mg/mL, 1 mL of phosphate buffer 0.15 M (pH 7.4), and 0.5 mL of 10% RBC suspension. In the control test tube, instead of a drug, only saline was added. Vitamin C was taken as a standard drug. All centrifuged tubes containing the reaction mixture were incubated at 56 °C for 30 min. After cooling and centrifugation (2500× *g*; 20 min), the percentage membrane stabilization activity was calculated as follows:

$$Inhibition\;of\;hemolysis\left(\%\right)=\frac{A1-A0}{A0}\times 100$$

where A0 is the absorbance of the control, and A1 is the absorbance in the presence of PSHT.

### 6.6. Effect of Sulfate Polysaccharide on Macrophages RAW264.7

#### 6.6.1. Cell Culture RAW264.7

The RAW 264.7 murine macrophage cell line (TIB-71) was obtained from the American Type Culture Collection (ATCC, Manassas, VA, USA). The cells were cultured in DMEM (Dulbecco’s Modified Eagle’s Medium, GIBCO, Life technologies, Carlsbad, CA, USA) supplemented with 10% fetal bovine serum (FBS; GIBCO, Life technologies, Carlsbad, CA, USA) and maintained at 37 °C in a humidified 5% CO_2_ incubator.

#### 6.6.2. Macrophages Viability Assay

A colorimetric assay for mitochondrial function evaluated the effects of sulfated polysaccharides on the viability of RAW264.7 cells. Briefly, cell viability was assessed by 3-(4.5-dime thylthiazol-2yl)-2.5-diphenyltetrazolium bromide (MTT) assay. Cells were seeded in 96-well plates (5000 cells/well) for 24 h and then incubated for 24 h in the presence of vehicle (control) and PSHT at different concentrations (15.6–5000 µg/mL). At the end of the treatments, 50 µL of MTT solution (1 mg/mL final) was added, and the cells were incubated for a further three hours at 37 °C. After that, the medium was removed, and 100 µL of dimethyl sulfoxide (DMSO) was added to each well to dissolve the formazan crystals. The optical density (OD) at 540 nm was measured with a microplate reader (MULTISCAN, Lab-systems).

Macrophage viability was expressed as a percentage of the viable cell number in treated cells relative to mock-treated cells (control). Macrophages proliferation was calculated using the following formula:

$$Macrophage\;proliferation\left(\%\right)=\frac{ODsample-ODblanc}{ODcontrol}\times 100$$

#### 6.6.3. Nitric Oxide Assay

The nitrite concentration was measured as an indicator of NO production according to the Griess reaction. In brief, RAW264.7 cells were seeded at a density of 1.5 × 10^5^ cells/well in 24-well plates. After 24 h incubation, cells were stimulated by LPS (1 μg/mL) for one hour before their treatments with vehicle or polysaccharides for 24 h. Subsequently, the supernatants (100 μL) were harvested and mixed with an equal volume of Griess reagent. Absorbance at 540 nm was measured by a microplate reader, and concentrations were calculated against a sodium nitrite standard curve.

### 6.7. Cytokines Quantification in Culture Supernatants

Mouse macrophage RAW264.7 cells were cultured at 1.5 × 10^5^/well in 24-well plates with DMEM medium supplemented with 10% FBS, and 100 U/mL penicillin, and 100 µg/mL streptomycin. After 24 h, the cells were stimulated with LPS (1 μg/mL) for one hour prior to their treatments with the appropriate doses of PSHT or vehicle for 24 h. The secreted cytokines in the supernatant of the RAW264.7 cells were determined using ELISA kits (GM-CSF: BD OptEIA, San Diego, CA, TNFα: DuoSet ELISA, R&D systems) according to the manufacturer’s instructions.

### 6.8. In Vivo PSHT Application

#### 6.8.1. Animals and Experimental Design

Male adult Wistar rats (150–200 g) were obtained from the Central Pharmacy of Tunis, Tunisia. All rats were maintained at room temperature (22–24 °C) on a 12 h light/dark cycle, with free access to food and water. The experimental procedures were carried out according to the Natural Health Institute of Health Guidelines for Animal Care and approved by the local Ethical Committee (Protocol nº 06.0003/23).

The experimental model of acute inflammation was created using carrageenan (CARR)-induced rat paw edema in vivo, as described by Winter et al. (1962) [92]. The CARR (0.1 mL of 1% *w*/*v*) was injected into the sub-plantar surface of the paw to produce acute inflammation.

In this experimental study, Wistar rats were randomized into four groups of six animals:

Group 1 (Controls): rats were mock-treated and received an isotonic saline solution (9%) (by oral gavage) used as a negative control.

Group 2 (CARR): rats were inflamed by CARR injection of 100 µL of CARR (1% suspension in 0.9% NaCl) and underwent no treatment.

Group 3 (CARR + DIC): rats were used as inflamed reference rats treated with DIC 20 mg/kg by oral gavage.

Group 4 (CARR + PSHT): rats were inflamed by CARR injection and treated with PSHT 20 mg/kg by oral gavage.

For each treated group, the size obtained at these various times (PT) was compared to that obtained before any treatment (P0). Percentile edema inhibition was determined according to the following formula:

$$Edema\;inhibition\left(\%\right)=\frac{Ac-Atest}{Atest}\times 100$$

#### 6.8.2. Sample Collection

After 5 h of treatment, blood samples were collected in EDTA tubes and were used for hematological analysis. Serums were collected from clotted blood after centrifugation at 3000 rpm for 15 min and were kept at −80 °C until analysis of biological parameters.

#### 6.8.3. Effect of PSHT and CARR on Hematological Parameters

Red blood cells (RBCs), white blood cells (WBCs), platelets (PLT), lymphocytes (LYM), hematocrit (HCT), hemoglobin concentration (Hb), mean cell hemoglobin (MCH), and mean cell hemoglobin concentration (MCHC) were evaluated by an electronic automate Coulter MAXM (Beckman Coulter, Inc., Fullerton, CA, USA) at Habib Bourguiba University Hospital of Sfax, Tunisia. The required peripheral blood smears were made on microscope slides and then preserved using water-free methanol. Then, a 1/10 diluted dye was used for Giemsa staining (Merck, Darmstadt, Germany). Finally, a 100× lens microscope was used to conduct the differential counting.

#### 6.8.4. Evaluation of Oxidative Stress Parameters in Skin and Erythrocytes

- Preparation of Extracts

Oxidative stress parameters were assessed in the skin (edema) and erythrocyte tissue homogenates diluted (10%, *w*/*v*) in phosphate buffer (pH 7.4) and centrifuged at 9000 rpm for 20 min. The resulting supernatants were used for oxidative stress assays.

- Determination of Skin and Erythrocyte Protein, Malondialdehyde, and Advanced Oxidation Protein Products Levels

Protein contents were determined using the Lowry method with bovine serum albumin as the standard [85]. Lipid peroxidation was measured in tissue homogenates [93]. AOPP levels were measured according to the method of Kayali et al. (2006) [94].

- Determination of Skin and Erythrocyte Enzymatic and Non-Enzymatic Anti-Oxidants

SOD activity was determined according to Beauchamp and Fridovich (1971) [95] and GPx activity was assessed according to the method of Flohe and Günzler (1984) [96].

#### 6.8.5. Histological Examination

Tissues from the individual animal’s paw (inflamed site) were collected for histopathological examination. All tissue samples were fixed in a 10% neutral buffered formalin solution, embedded in paraffin wax, cut into 5 mm thick sections, and stained with hematoxylin–eosin. Slides were photographed with a Canon camera connected to an optical microscope (Olympus Inc., Tokyo, Japan).

### 6.9. Computational Study

The computational approach focused on the assessment of both binding affinities and molecular interactions of the polysaccharide units (arabinose, d-fructose, d-galactose, d-glucose, d-ribose, xylose, glucuronic acid, and mannose) with two targeted receptors: COX-2 and TNF-α. Both monosaccharides and receptors were processed and then subjected to a CHARMm force field for the complex formations, as previously reported [78,80,82]

### 6.10. Statistical Analysis

Statistical analyses were performed using ANOVA analysis at a *p* level ≤ 0.05 with the Graph Pad Prism version. A standard deviation at the 95% confidence level was used.

## 7. Conclusions

The crude polysaccharide PSHT extracted from green seaweed *Halimeda tuna* exhibits promising anti-oxidant and anti-hemolytic activity. In addition, PSHT had a significant regulatory effect on the RAW264.7 macrophages, with no toxic effect observed and promoting cell growth ability. As an important indicator of the immune response, the NO production by RAW264.7 cells was significantly regulated after treatment with LPS. Meanwhile, the cytokines GM-CSF and TNF-α were significantly decreased after treatment with LPS and PSHT. In addition, PSHT presented a potent anti-inflammatory activity confirmed by reduced inflammatory symptoms like edema and oxidative stress in both skin and erythrocytes in vivo. Our computational results supported the experimental findings and demonstrated the beneficial effects of the crude polysaccharides extracted from *H. tuna*. As a natural polysaccharide, the PSHT from *H. tuna* could be a promising alternative for biotechnological applications, especially those related to immunomodulatory potential and human health.

## Figures and Tables

**Table 1:** Chemical analysis of PSHT.

Parameters	PSHT
Yield (%)	15.9 ± 0.5
Total sugar (%)	70.0 ± 0.3
Total sulfate (%)	3.6 ± 0.3
Uronic acid (%)	17.2 ± 0.0
Protein (%)	1.9 ± 0.2

Values are expressed as mean ± SEM (*n* = 3).

**Table 2:** Percentage of edema inhibition in CARR+DIC and CARR + PSHT groups.

Groups	1H	2H	3H	4H	5H
CARR + DIC	4.1 ± 1.2%	25 ± 3.0%	50 ± 1.0%	73.1 ± 0.7%	88.9 ± 0.7%
CARR + PSHT	3.1 ± 0.4%	20.9 ± 2.4%	48.6 ± 0.5%	69.2 ± 0.2%	88.7 ± 0.3%

CARR + DIC: rats injected with CARR and gavaged with DIC 20 mg/kg; CARR + PSHT: rats injected with CARR and gavaged with PSHT 20 mg/kg. Values are expressed as means ± S.D. for six animals in each group.

**Table 3:** Effect of treatment with CARR, PSHT, and DICL on hematological parameters.

	Controls	CARR *	CARR + DIC ^#^	CARR + PSHT ^#^
WBC	10.7 ± 0.1	15.3 ± 01 ***	11.4 ± 0.1 ^###^	11.7 ± 0.1 ^###^
RBC	7.7 ± 0.1	6.6 ± 0.2 ***	7.6 ± 0.1 ^###^	7.6 ± 0.0 ^###^
LYM	42.2 ± 1.9	62.6 ± 1.6 ***	45.4 ± 1.7 ^###^	45.1 ± 0.7 ^###^
PLT	983 ± 8.7	1085.8 ± 21.9 ***	944.3 ± 12.5 ^###^	989.8 ± 45.1 ^###^
Hb	13.2 ± 0.2	10.5 ± 0.4 ***	13.2 ± 0.3 ^###^	12.9 ± 0.3 ^###^
MCHC	31.2 ± 0.3	31.3 ± 0.2	31.4 ± 0.0	31.3 ± 0.2
MCH	18.1 ± 0.3	18.1 ± 0.2	18.2 ± 0.3	18.2 ± 0.4
Ht	35.5 ± 1.6	36.1 ± 1.4	35.8 ± 1.9	36.0 ± 0.8

Values are expressed as mean ± SEM (*n* = 6) in each group. Controls: rats treated with NaCl 0.9%; CARR: rats treated with carrageenan 1%; CARR + DICL: rats injected with CARR and gavaged with DIC 20 mg/kg; CARR + PSHT: rats injected with CARR and gavaged with PSHT 20 mg/kg. Values are expressed as means ± S.D. for 6 animals in each group. * compared to control; ^#^ compared to CARR. *** *p* < 0.001, and ^###^ *p* < 0.001. WBC: white blood cells, RBC: red blood cells, LYM: lymphocytes, PLT: Platelets, Hb: hemoglobin, MCHC: mean corpuscular hemoglobin concentration, MCH: mean corpuscular hemoglobin, Ht: hematocrit.

**Table 4:** Lymphocytes, neutrophils, monocytes, and eosinophils cells infiltration in carrageenan-induced paw edema.

	Controls	CARR *	CARR + DIC ^#^	CARR + PSHT ^#^
Neutrophils (%)	44 ± 1.3	50 ± 1.3 **	45 ± 0.7 ^##^	44 ± 0.2 ^##^
Monocytes (%)	01 ± 0.3	02 ± 0.1	03 ± 0.1	06 ± 0.1 ^##^
Eosinophils (%)	01 ± 0.1	00	02 ± 0.1	02 ± 0.1
Lymphocytes (%)	42.2 ± 0.3	48 ± 0.7 **	43.2 ± 0.3 ^##^	44 ± 1.3 ^##^

Values are expressed as mean ± SEM (*n* = 6) in each group. Controls: rats treated with NaCl 0.9%; CARR: rats treated with carrageenan 1%; CARR + DICL: rats injected with CARR and gavaged with DIC 20 mg/kg; CARR + PSHT: rats injected with CARR and gavaged with PSHT 20 mg/kg. Values are expressed as means ± S.D. for 6 animals in each group. * compared to control; ^#^ compared to CARR., ** *p* < 0.01 and ^##^ *p* < 0.01.

**Table 5:** Levels of serum proteins in controls and treated rats with CARR, PSHT, and DICL.

	Total Proteins	Albumin	Alpha1-Globulin	Beta1-Globulin	Gamma-Globulin
Controls (g/dL)	54.6 ± 0.2	22.1 ± 0.4	7.1 ± 0.3	7.4 ± 0.2	7.2 ± 0.0
CARR * (g/dL)	63.6 ± 1.4 ***	18.0 ± 0.3 ***	10.8 ± 0.1 ***	8.3 ± 0.3 ***	7.8 ± 0.1 ***
CARR + DIC ^#^ (g/dL)	55.1 ± 0.6 ^###^	20.7 ± 0.7 ^###^	7.2 ± 0.2 ^###^	7.4 ± 0.0 ^###^	7.2 ± 0.0 ^###^
CARR + PSH ^#^ (g/dL)	56.1 ± 0.1 ^###^	21.1 ± 0.6 ^###^	7.4 ± 0.4 ^###^	7.3 ± 0.2 ^###^	7.1 ± 0.1 ^###^

Values are expressed as mean ± SEM (*n* = 6) in each group. Control: rats treated with NaCl 0.9%; CARR: rats treated with carrageenan 1%; CARR + DICL: rats injected with CARR and gavaged with DIC 20 mg/kg; CARR + PSHT: rats injected with CARR and gavaged with PSHT 20 mg/kg. Values are expressed as means ± S.D. for 6 animals in each group. * compared to control; ^#^ compared to CARR. *** *p* < 0.001, and ^###^ *p* < 0.001.

**Table 6:** Binding affinity, conventional hydrogen bonds, and interacting residues of the monosaccharides (arabinose, d-fructose, d-galactose, d-glucose, d-ribose, xylose, glucuronic acid, and mannose) complexed with COX-2 and TNF-α receptors.

Monosaccharide (Ligand)	Target Receptor	Intermolecular Interactions			
		Binding Affinity (kcal/mol)	No. Closest Interacting Residues	Closest Interacting Residue (Distance, Å)	No. H-Bonds
Arabinose	COX-2	−5.8	4	Asn39 (2.06)	5
	TNF-α	−4.9	4	Glu135 (1.16)	5
D-fructose	COX-2	−6.6	7	Asn34 (2.04)	6
	TNF-α	−4.9	3	Ile136 (1.91)	7
D-galactose	COX-2	−6.4	6	Glu465 (1.92)	8
	TNF-α	−4.9	5	Asn46 (1.85)	5
D-glucose	COX-2	−6.3	5	Gln461 (2.15)	7
	TNF-α	−4.5	4	Leu142 (1.97)	4
D-ribose	COX-2	−5.9	4	Gly45 (1.88)	6
	TNF-α	−4.3	3	Glu135 (1.96)	5
Xylose	COX-2	−6.1	4	Ser121 (1.90)	4
	TNF-α	−4.6	3	Leu26 (2.10)	4
Glucuronic acid	COX-2	−6.5	5	Thr212 (2.06)	6
	TNF-α	−5.1	4	Gln25 (2.15)	4
Mannose	COX-2	−6.3	4	Asn571 (1.88)	5
	TNF-α	−4.8	4	Ile136 (2.07)	5

## Data Availability

The data presented in this study are available on request from the corresponding author.
